# Wavy Floating Greater Omentum Findings Are Useful for Differentiating the Etiology of Fetal Ascites

**DOI:** 10.3390/diagnostics11020326

**Published:** 2021-02-17

**Authors:** Takashi Iizuka, Masanori Ono, Rena Yamazaki, Kyosuke Kagami, Yusuke Mitani, Seisho Sakai, Hiroshi Fujiwara

**Affiliations:** 1 Department of Obstetrics and Gynecology, Graduate School of Medical Science, Kanazawa University, Kanazawa 920-8640, Japan; masanori@med.kanazawa-u.ac.jp (M.O.); rena76jp@ybb.ne.jp (R.Y.); ktykkn@yahoo.co.jp (K.K.); fuji@med.kanazawa-u.ac.jp (H.F.); 2 Department of Pediatrics, Graduate School of Medical Science, Kanazawa University, Kanazawa 920-8640, Japan; pyusuke35018@staff.kanazawa-u.ac.jp; 3 Department of Pediatric Surgery, Graduate School of Medical Science, Kanazawa University, Kanazawa 920-8640, Japan; s-sakai@staff.kanazawa-u.ac.jp

**Keywords:** greater omentum, fetal ascites, prenatal diagnosis, meconium peritonitis

## Abstract

The greater omentum is an apron-like peritoneal mesothelial sheet that was described by ultrasound as a floating fluid-filled viscus in ascites during the fetal period. To examine the association between the etiology of fetal ascites and ultrasound findings of the greater omentum, a retrospective study was conducted. Ultrasound findings of fetal omentum were defined as follows: (1) a cyst-like shape with a thin membrane observed as wavy in the ascites, (2) beside the stomach and below the liver, and (3) no blood flow noted on color Doppler. Eleven pregnancies had fetal ascites. A fetal greater omentum was confirmed in eight cases in which ascites were caused by non-peritonitis: fetal hydrops (*n* = 4), congenital cytomegalovirus infection (*n* = 2), idiopathic chylous ascites (*n* = 1), and unknown cause (*n* = 1). Of these eight cases, no abdominal surgical management was required in three live babies. However, a fetal greater omentum was not confirmed in all three cases of meconium peritonitis. It was suggested that the finding of the greater omentum can be an important clue for estimating the pathophysiological etiology of fetal ascites and helping with postnatal management. It should be reasonable to add the finding of the greater omentum to the detailed ultrasound examination checklist.

## 1. Introduction

Fetal ascites is diagnosed as the presence of fluid in the abdominal cavity by ultrasound. The etiology of fetal ascites includes obstruction of venous return by a space-occupying lesion in the thorax, cardiac failure, fetal anemia, congenital infection such as cytomegalovirus and parvo B19 virus, and meconium peritonitis [1]. To identify the etiology of fetal ascites, a careful search including maternal infection screening and detailed fetal ultrasound should be performed [2]. It can be accompanied by fetal hydrops with subcutaneous edema, pleural effusion, and pericardial effusion. Even in the absence of fetal hydrops, isolated fetal ascites can be the first sign of hydrops [3]. Since a gastrointestinal cause often necessitates surgical intervention early after birth [3], it is important to assess whether the cause of ascites is gastrointestinal, such as meconium peritonitis. However, it is difficult to differentiate the cause of isolated fetal ascites prenatally [3,4,5,6], and invasive paracentesis should be performed to evaluate the characteristics of ascites [2].

The greater omentum is an apron-like peritoneal mesothelial sheet that extends from the greater curvature of the stomach. Barry et al. (1982) described the fetal greater omentum on ultrasound as a loculated area of fluid or a fluid-filled viscus in ascites [7]. They suggested that if the amount of ascites is large enough to be present in both the omentum bursa (lesser sac) and peritoneal space (greater sac), the greater omentum will show an outline of the membrane in the ascites. Since the greater omentum is not identified in cases of child or adult ascites [7], it is speculated that its finding depends on fetal development. Moreover, it was speculated that the greater omentum cannot be observed in ascites with meconium peritonitis (T. Minamitani, T. Funakoshi, Personal communication, 24 August 2020), because the greater omentum should form an adhesion to the contaminated areas in peritonitis [8]. However, no published studies or reports discuss the association between ultrasound findings of the fetal greater omentum and the etiology of fetal ascites. In this study, we examined the relationship between fetal greater omentum findings and the etiology of fetal ascites.

## 2. Materials and Methods

A retrospective study of all pregnant women managed between April 2014 and March 2020 in the Department of Obstetrics and Gynecology at Kanazawa University Hospital was conducted. Ultrasound examinations were performed using a GE Voluson E8 with a 2-5-MHz transducer and Aloka Prosound Alpha 6 with a 2-6-MHz transducer. The inclusion criteria were pregnancies presenting with significant fetal ascites. Significant fetal ascites was defined as the presence of fluid in the abdominal cavity accompanied by an increased abdominal circumference [9]. Based on a previous report [7], the ultrasound findings of fetal greater omentum were defined as follows: (1) a cyst-like shape with a thin membrane observed as wavy in the ascites, (2) beside the stomach and below the liver, and (3) no blood flow noted on color Doppler. A follow-up ultrasound was performed to assess the amount of ascites, examine the growth of the fetus, and detect any other abnormalities. The prenatal workup for ascites included the maternal blood type and antibody status, and infection screening including parvovirus B19, cytomegalovirus, hepatitis B and C, herpes simplex, rubella, and toxoplasmosis. The need for chromosomal studies of the fetus was suggested in all cases, but consent for testing was not obtained. The method and timing of delivery were determined in individual cases. After delivery, the cause of ascites was assessed based on the characteristics of ascitic fluid including cytology, autopsy, surgical findings, and testing for infections in newborns. Follow-up of infants was available through the Neonatal Intensive care unit. This study was approved by the Medical Ethics Committee of Kanazawa University.

## 3. Results

### 3.1. Summary of Fetal Ascites Cases

Eleven pregnancies had significant fetal ascites accompanied by an increased abdominal circumference during the study period (Table 1). Fetal ascites was diagnosed between 18 and 39 weeks of gestation. A fetal greater omentum was observed in eight cases (cases 1–8), and it was unobserved in three cases (case 9–11).

### 3.2. Fetal Greater Omentum with Ascites

**Figure 1:**
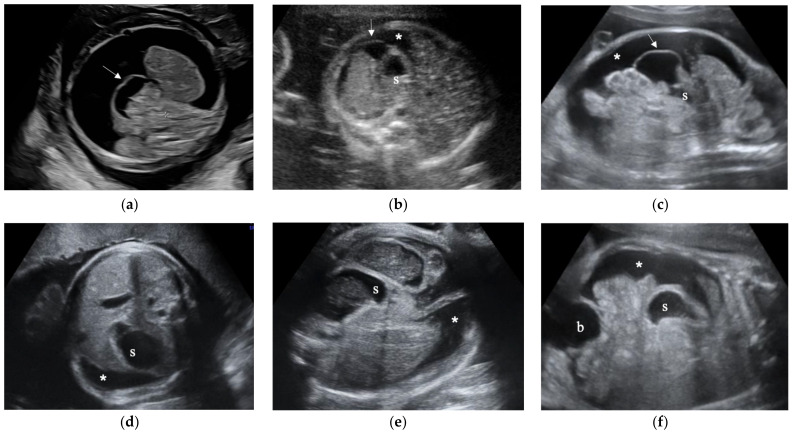
Ultrasound images of fetal ascites. (**a**) case 4, 22 weeks of gestation, (**b**) case 6, 26 weeks of gestation, (**c**) case 7, 28 weeks of gestation, (**d**) case 9, 31 weeks of gestation, (**e**) case 10, 39 weeks of gestation. (**f**) case 11, 28 weeks of gestation. Asterisk (*): ascites, arrow: greater omentum, s: stomach, b: bladder.

There were four cases of fetal hydrops with ascites (cases 1–4). In case 2, the lymphocyte-dominated ascites was identified from the stillborn baby. In case 3 and case 4, the cause of fetal hydrops was compression of the inferior vena cava associated with an intrathoracic lesion and hyperinflated lungs by laryngeal atresia, respectively (Figure 1a) and congenital pulmonary airway malformation, respectively. In both cases, the ascites was improved and the greater omentum was not observed at 30 weeks. There were four cases of isolated ascites with a floating greater omentum (cases 4–8). Cases 5 and 6 (Figure 1b) were diagnosed with congenital cytomegalovirus infection. Maternal Cytomegalovirus(CMV)-IgM antibody was positive in case 6 but negative in case 5. CMV-DNA was detected in the urine of both newborns. In case 6, the fetal ascites resolved spontaneously, and the greater omentum was not observed at 30 weeks of gestation. Case 7 (Figure 1c) resulted in intrauterine fetal death of unknown cause at 32 weeks. The greater omentum was observed by ultrasound until just before the fetal death. At autopsy, there was no abnormality of the gastrointestinal tract, and the lymphocyte-dominated chylous ascites was confirmed.

### 3.3. Fetal Greater Omentum Was Not Observed in Three Cases of Meconium Peritonitis

A fetal greater omentum was not observed in all three cases of meconium peritonitis (cases 9–11, Figure 1d–f). None of the three cases showed fetal hydrops. Polyhydramnios was observed in cases 9 (Figure 1d) and 11 (Figure 1f). In case 10, the fetal dilated small intestine was observed, and intestinal atresia was suspected. At 39 weeks of gestation, fetal ascites suddenly appeared (Figure 1e), and an emergency cesarean section was performed due to a non-reassuring fetal status. After birth, the neonate underwent emergency surgery and was diagnosed with meconium peritonitis by midgut volvulus with intestinal malrotation and atresia. In case 11, fetal ascites resolved spontaneously, and intra-abdominal calcification was observed during the third trimester of pregnancy (Figure 1f).

## 4. Discussion

In this study, we could observe a fetal omentum in eight cases, and at least six of these cases were attributed to non-peritonitis based on autopsy and clinical findings. However, we did not observe a fetal greater omentum in all three cases of meconium peritonitis.

**Figure 2:**
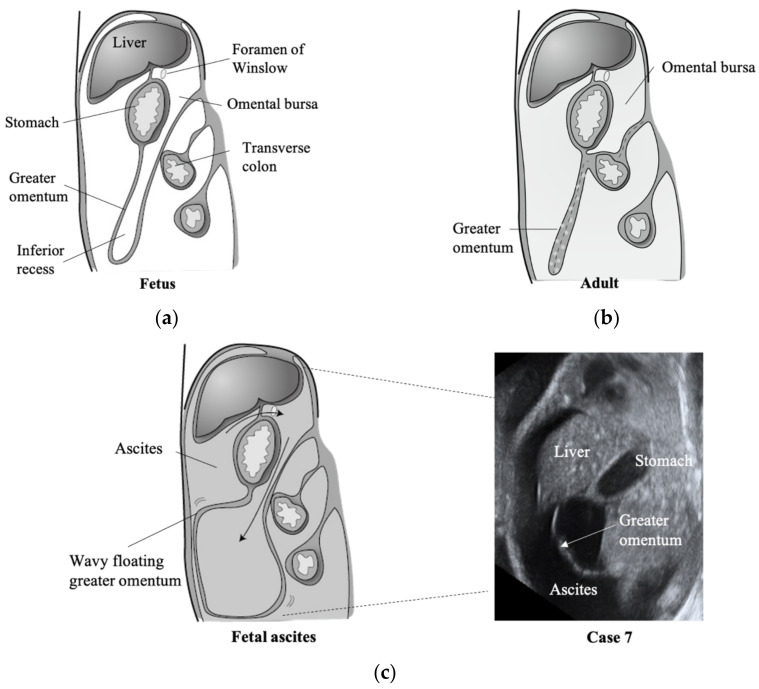
Schematic diagram of the greater omentum in an adult (**a**), fetus (**b**), and fetus with ascites (**c**). The parts where the membrane is fused are shown by dashed lines in (**b**). Arrows in (**c**): ascites enters the omental bursa through the foramen of Winslow into the inferior recess.

We considered that the finding of a floating greater omentum in ascites is associated with omentum development during the fetal period. The greater omentum forms in the 12th week of gestation [10]. The omentum is a folded double layer of peritoneum connecting to the gastric greater curvature, and the omental bursa reaches as low as the free margin of the greater omentum, which is described as an inferior recess (Figure 2a). During fetal development, the dorsal mesentery of the transverse colon and dorsal part of the mesogastrium fuse to become the transverse mesocolon. The inferior recess is obliterated by fusion of the anterior and posterior sheet of the greater omentum, forming the lower border of the omental bursa [10,11]. In adults, the vertical extent of the omental bursa is limited due to the fusion of the greater omentum (Figure 2b), and only the omental bursa can be identified in ascites [11]. During the fetal period, a large amount of ascites fluid will flow into the inferior recess of the omental bursa through the foramen of Winslow, and a floating greater omentum in ascites can be demonstrated (Figure 2c).

We did not observe the greater omentum in fetal ascites caused by meconium peritonitis. We presume that the unobserved greater omentum was due to the omentum adhesion by inflammation. The omentum has several functions during peritonitis: absorption and clearance of bacteria and foreign material from the peritoneal cavity [12], supplying leukocytes to the peritoneal cavity [13], and adhering to areas of contamination. One of the functions of the greater omentum is to adhere to seal off contaminated local areas and inhibit the spread of inflammation [8]. Moreover, the adherence of the omentum promotes angiogenesis and fibroplasia, which has a beneficial influence on healing [14]. The omentum will cover the contaminated area forming dense adhesion, and it can be recognized as omental masses in meconium peritonitis [15]. If the greater omentum adheres by meconium peritonitis during the fetal period, ascites fluid cannot flow into the inferior recess, and it cannot be detected on ultrasound as a fluid-filled viscus.

We assess the diagnostic utility of greater omentum by discussing case 9. In this case, fetal ascites and polyhydramnios were observed, but the fetal greater omentum was not observed. Although polyhydramnios indicates gastrointestinal obstruction associated with meconium peritonitis, it is also observed in non-peritonitis causes. Since the greater omentum was not observed, it was expected that the etiology of the fetal ascites was inflammation of the abdominal cavity. Therefore, we consulted the pediatric surgery team and prepared for postnatal management, which allowed us to treat the newborn baby smoothly. From this case, we suggest that the finding of the greater omentum can be an important clue for estimating the pathophysiological etiology of fetal ascites and helping with postnatal management. It should be reasonable to add the finding of the greater omentum to the detailed ultrasound examination checklist.

There were limitations of this study. In this study, we observe the greater omentum from 18 to 32 weeks but not after that because of the spontaneous remission of ascites. Since the fusion between the layers of the greater omentum is considered to have occurred at full-term pregnancy [10,11], later in pregnancy, the greater omentum with ascites is less visible. In our cases of meconium peritonitis, the number of gestational weeks at the onset of ascites was later (28 to 39 weeks) compared with other causes (18 to 28 weeks). Especially in case 11, the greater omentum might have been fused. Changes in ultrasound findings of the greater omentum in late pregnancy should be considered. In addition, the number of meconium peritonitis cases was only three in this study. We need to accumulate more cases to verify the association between findings of the greater omentum and causes of ascites.

In conclusion, fetal greater omentum was observed as a wavy cyst-like shape in the ascites, and it was only observed in non-peritonitis fetal ascites cases. It was speculated that unobserved greater omentum in meconium peritonitis cases was due to the omentum adhesion. Findings of the greater omentum on noninvasive ultrasound are useful for differentiating the cause of ascites and helping with postnatal management. Further study is needed to verify the association between findings of the greater omentum and causes of ascites.

## Figures and Tables

**Table 1:** Summary of 11 cases of fetal ascites.

Case	Gestational Weeks at Diagnosis	Greater Omentum Finding	Associated and Additional Ultrasound Findings	Fetal Hydrops	Outcome (Weeks of Delivery)	Etiology and Associated Disorder of Ascites
1	21w	yes	Omphalocele, Cystic hygroma	yes	IUFD (21w)	Fetal malformations, Non-immune hydrops
2	20w	yes	Pleural effusion, Giant placental hematoma	yes	IUFD (23w)	Non-immune hydrops, Chylous ascites
3	18w	yes	Laryngeal atresia, PA/VSD, Dilated loops of bowel, Polyhydramnios *Ascites remission (32w)	yes	Stillbirth (35w)	Compression of inferior vena cava by hyperinflated lungs. Meconium peritonitis was not found at autopsy
4	18w	yes	CPAM, Polyhydramnios *Ascites remission (30w)	yes	Live birth, NVD (41w)	Compression of inferior vena cava by CPAM
5	23w	yes	Umbilical cord cysts	no	Live birth, Emergency C/S due to CAM (28w)	Congenital CMV infection (maternal CMV–IgM-negative), Chylous ascites
6	26w	yes	Neck and abdominal cysts, Polyhydramnios *Ascites remission (30w)	no	Live birth, Emergency C/S due to NRFS (37w)	Congenital CMV infection (maternal CMV–IgM-positive)
7	28w	yes	Polyhydramnios	no	IUFD (32w)	Idiopathic (chylous ascites)
8	18w	yes	Omphalocele, Cleft lip, and palate	no	TOP (19w)	Fetal malformations
9	31w	no	Polyhydramnios	no	Live birth, Emergency C/S due to NRFS (32w)	Meconium peritonitis, Small bowel atresia
10	39w	no	Dilated loops of bowel	no	Live birth, Emergency C/S due to NRFS (39w)	Meconium peritonitis, Midgut volvulus with intestinal malrotation and atresia
11	28w	no	Abdominal calcification, Dilated loops of bowel, Polyhydramnios *Ascites remission (33w)	no	Live birth, NVD (38w)	Meconium peritonitis, Intussusception

IUFD = Intrauterine fetal demise; TOP = Termination of pregnancy; PA/VSD = Pulmonary atresia with ventricular septal defect; CPAM = Congenital pulmonary airway malformation; CAM = Chorioamnionitis; CMV = Cytomegalovirus; C/S = Cesarean section; NRFS = Non-reassuring fetal status; NVD = normal vaginal delivery.

## Data Availability

The authors confirm that the data supporting the findings of this study are available within the article.
